# Relationship between Pre-Implant Interleukin-6 Levels, Inflammatory Response, and Early Outcome in Patients Supported by Left Ventricular Assist Device: A Prospective Study

**DOI:** 10.1371/journal.pone.0090802

**Published:** 2014-03-04

**Authors:** Raffaele Caruso, Luca Botta, Alessandro Verde, Filippo Milazzo, Irene Vecchi, Maria Giovanna Trivella, Luigi Martinelli, Roberto Paino, Maria Frigerio, Oberdan Parodi

**Affiliations:** 1 CNR Institute of Clinical Physiology, CardioThoracic and Vascular Department, Niguarda Ca’ Granda Hospital, Milan, Italy; 2 CardioThoracic and Vascular Department, Niguarda Ca’ Granda Hospital, Milan, Italy; 3 CNR Institute of Clinical Physiology and University of Pisa, Pisa, Italy; Virginia Commonwealth University Medical center, United States of America

## Abstract

**Purpose:**

The immune response is crucial in the development of multi-organ failure (MOF) and complications in end-stage heart failure patients supported by left ventricular assist device (LVAD). However, at pre-implant, the association between inflammatory state and post-LVAD outcome is not yet clarified. Aim of the study was to assess the relationship among pre-implant levels of immune-related cytokines, postoperative inflammatory response and 3-month outcome in LVAD-patients.

**Methods:**

In 41 patients undergoing LVAD implantation, plasma levels of interleukin (IL)-6, IL-8, crucial for monocyte modulation, and urine neopterin/creatinine ratio (Neo/Cr), marker of monocyte activation, were assessed preoperatively, at 3 days, 1 and 4 weeks post-LVAD. MOF was evaluated by total sequential organ failure assessment (tSOFA) score. Intensive care unit (ICU)-death and/or post-LVAD tSOFA ≥11 was considered as main adverse outcome. Length of ICU-stay, 1 week-tSOFA score, hospitalisation and 3-month survival were considered additional end-points.

**Results:**

During ICU-stay, 8 patients died of MOF, while 8 of the survivors experienced severe MOF with postoperative tSOFA score ≥11. Pre-implant level of IL-6 ≥ 8.3 pg/mL was identified as significant marker of discrimination between patients with or without adverse outcome (OR 6.642, 95% CI 1.201-36.509, *p* = 0.030). Patients were divided according to pre-implant IL-6 cutoff of 8.3 pg/ml in A [3.5 (1.2–6.1) pg/mL] and B [24.6 (16.4–38.0) pg/mL] groups. Among pre-implant variables, only white blood cells count was independently associated with pre-implant IL-6 levels higher than 8.3 pg/ml (OR 1.491, 95% CI 1.004–2.217, *p* = 0.048). The ICU-stay and hospitalisation resulted longer in B-group (*p* = 0.001 and *p* = 0.030, respectively). Postoperatively, 1 week-tSOFA score, IL-8 and Neo/Cr levels were higher in B-group.

**Conclusions:**

LVAD-candidates with elevated pre-implant levels of IL-6 are associated, after intervention, to higher release of monocyte activation related-markers, a clue for the development of MOF, longer clinical course and poor outcome.

## Introduction

In an era characterised by lack of suitable organs for transplantation, continuous flow left ventricular assist devices (CF-LVADs) bridge patients with end-stage heart failure (ESHF) to transplantation (BTT), to further decision (BTD), or to recovery, or are implanted as destination therapy (DT) [1]–[3]. Despite progressive improvements in technologies, intraoperative and perioperative management, favourable outcomes still depend on proper patient selection and strategic timing of implantation. Indications, absolute or relative contraindications are not universally accepted and contrasting data have been published [1]–[4]. With worsening of clinical status of ESHF patients, increase the need for a mechanical circulatory support (MCS) as the peri-operative risk, resulting in a greater exertion in managing the timing of implant [1]–[4]. Indeed, in many centers, LVAD implantation is anticipated, preferably before that the patient experiences hemodynamic collapse [5].

Adverse outcomes and development of multi-organ failure (MOF) in LVAD-patients are related to the activation of systemic inflammation, although mechanisms underlying the multi-organ deterioration remain still poorly understood [6]. The levels of interleukin (IL)-6 and IL-8, crucial cytokines for the activation of systemic inflammatory pathways, and neopterin, a marker reflecting monocyte activation, are found to increase after LVAD implantation, particularly in patients affected by MOF [7], the main cause of death during the early phase of MCS [8]. Moreover, pre-implant levels of IL-6 have been associated with hemodynamic status, as defined by Interagency Registry for Mechanically Assisted Circulatory Support (INTERMACS) profiles, with higher levels in patients presenting critical INTERMACS profiles [9]. Since the signal pathways, IL-6-dependent, and specific monocyte attracting chemokines, such as IL-8, are proposed as crucial triggers in controlling monocyte activation, an important condition in the development of MOF and of haemostatic complications [10], it can be assumed that they play a critical role in affecting outcomes during the early phase of LVAD support. The aims of this study were to assess whether preoperative IL-6, IL-8 and neopterin levels affect postoperative inflammatory response and short-term (3 months) outcomes in LVAD-recipients.

## Methods

### Patients

From January 2005 to February 2012, 56 VAD implantations have been performed in ESHF-patients at our institution. Nothing was changed in our VAD peri-operative management protocol along these years. Patients with a diagnosis of myocarditis or undergoing MCS with a short term device (intra aortic balloon pump, Impella Recover, peripheral or intra-thoracic extracorporeal membrane oxygenation), with a pulsatile or biventricular VAD were excluded as well as patients undergoing concomitant cardiac procedures. One patient listed for a long-term CF-LVAD, requiring unplanned extra-corporeal membrane oxygenation (ECMO) support for sudden circulatory failure before LVAD support, was included. Patients with acute cardio-circulatory failure, treated with ECMO as BTD and later treated with complex devices (long-term, intra-corporeal, continuous axial or centrifugal flow LVADs), were not included.

Forty-one patients complying the selection criteria according to guideline indications for mechanical support [11], were definitively enrolled for this study.

Twenty chronic HF (CHF) patients, matched for age, sex, diagnosis and NYHA classes with LVAD-candidates, were enrolled to compare the cytokine levels between chronic state and end-stage of HF disease.

### Ethics Statement

This study complied with the principles of the Declaration of Helsinki. The study protocol has been approved by the Ethics Committee of Niguarda Ca’ Granda Hospital (Milan, Italy) and a signed informed consent has been obtained by all participating patients.

### Study design and assays

Baseline demographics, operative characteristics and postoperative details were collected for all patients. Trans-thoracic and or trans-esophageal echocardiography was pre-operatively performed. Hemodynamic data were assessed pre-operatively and then daily, up to a maximum of 1 week, by means of a pulmonary artery Swan-Ganz catheter. MOF was monitored pre-operatively and up to a maximum of 2 weeks calculating the total Sequential Organ Failure Assessment (tSOFA) score. The SOFA system, used for predicting intensive care unit (ICU)-mortality [12], is a daily score from 0 to 4 assigned in proportion to the severity of functional deterioration for each of 6 individual organ systems (cardiovascular, respiratory, hepatic, renal, neurologic, and hemocoagulative). The tSOFA score was calculated by adding the scores for each of the organ systems during the first post-operative weeks [12].

After the operation, right heart dysfunction was diagnosed in the presence of inotropic equivalent >10 and/or right atrial pressure >10 mm Hg [13]. Renal function was assessed by estimated glomerular filtration rate using the abbreviated MDRD formula [14].

The combination of postoperative tSOFA score ≥11 [15] and/or ICU-death was taken into account as main composite adverse outcome.

The following end-points were also considered: tSOFA score at 1 week, length of ICU stay, hospitalisation, and 3-month survival.

### Inflammatory parameters

In LVAD-patients, plasma IL-6, IL-8 levels, and urine neopterin levels, a known marker of monocyte activation [16], were measured pre-operatively and at 3, 7 and 30 days after intervention. In all patients, the blood and urine samples were collected pre-operatively in a range limited to 24 hours before cardiopulmonary bypass induction. Plasma IL-6 and IL-8 levels were measured according to the method of the manufacturer of the enzyme-linked immunosorbent assays (R&D Systems, Minneapolis, MN), whereas urinary neopterin levels were measured by an isocratic HPLC method as previously described and normalized by the urine creatinine concentrations as neopterin/creatinine (Neo/Cr) ratio [6].

### Statistical Analysis

Data are expressed as median and interquartile range (I-III) or frequency (percentage).

Receiver-operating characteristics (ROC) curve and the area under curve (AUC) was performed to determine the best cut-off that discriminate patients with or without adverse outcome. The associations between composite outcome, categorical IL-6 variable and clinical or biochemical parameters was assessed by univariable logistic regression analysis; significant variables (*p*<0.10) were then entered into a multivariable logistic regression model. Results are presented as odds ratio (OR) and their 95% confidence interval (CI). Differences between groups were assessed by Student T test or nonparametric Mann-Whitney test for continuous variables and by Chi-square or Fisher exact test for categorical variables. Differences of time-course of biochemical and clinical variables between groups were assessed by nonparametric Friedman test followed by Wilcoxon post-hoc test. A two-tailed *p*-value <0.05 was considered statistically significant.

## Results

### Patient characteristics and postoperative outcome

Clinical data of candidates to LVAD implantation and operative characteristics are described in Table 1. Twenty-five patients were treated for a dilated cardiomyopathy. Twenty-eight patients were in NYHA class IV, while the other patients were in NYHA class III. Preoperative intravenous inotropes were used in 25 patients while intra-aortic balloon pump in 13. CF-LVADs were implanted in 35 patients as BTT, in 3 patients as BTD and in 3 patients as DT.

**Table 1:** Univariable logistic regression analysis of variables associated to patient group with composite adverse outcome.

	All Cases	Without composite outcome	With composite outcome	*P*
	(n = 41)	(n = 25)	(n = 16)	
Age, yrs	55 (47–61)	54 (46–58)	56 (47–64)	0.267
Male gender, n (%)	37 (90)	24 (96)	13 (81)	0.155
Etiology, n (%)				0.873
IDC	25 (61)	15 (60)	10 (63)	
ICM	16 (39)	10 (40)	6 (37)	
NYHA class, n (%)				0.960
III	13 (32)	8 (32)	5 (31)	
IV	28 (68)	17 (68)	11 (69)	
INTERMACS, n (%)				
1	11 (27)	6 (24)	5 (31)	(Reference)
2	9 (22)	6 (24)	3 (19)	0.583
3+4	21 (51)	13 (52)	8 (50)	0.688
Pre-implant data				
LVEF, %	22 (18–25)	23 (18–25)	20 (18–25)	0.263
LVEDV, ml	260 (188–315)	260 (190–330)	248 (175–304)	0.426
LVEDD, mm	70 (64–77)	70 (64–78)	66 (64–76)	0.274
CI, L/min/m^2^	1.68 (1.37–2.02)	1.76 (1.53–2.10)	1.49 (1.33–1.72)	0.110
RAP, mmHg	6 (4–10)	5 (3–6)	9 (5–14)	0.035
PCWP, mmHg	26 (18–30)	24 (15–30)	28 (24–33)	0.186
MAP, mmHg	75 (69–83)	78 (71–84)	73 (68–82)	0.373
Treatments, n (%)				
ACEi+ATII	29 (74)	18 (75)	11 (73)	0.908
Beta-Blocker	24 (65)	16 (70)	8 (57)	0.445
Statins	12 (32)	7 (32)	5 (33)	0.923
Diuretics	32 (82)	20 (83)	12 (80)	0.792
Inotropic	25 (61)	15 (60)	10 (67)	0.923
Inotropic equivalent, n	8 (3–10)	8 (3–10)	8 (4–12)	0.816
IABP, n (%)	13 (32)	7 (28)	6 (38)	0.525
INR	1.20 (1.08–1.38)	1.12 (1.03–1.30)	1.21 (1.15–1.42)	0.370
WBC, 10^9^/L	8.4 (6.5–10.4)	8.7 (7.1–11.5)	8.2 (5.7–8.7)	0.308
Lactate, nmol/l	1.00 (0.75–1.65)	1.00 (0.70–1.60)	1.00 (0.78–1.88)	0.402
eGFR, ml/min/1.73 m^2^	80 (58–107)	85 (75–114)	64 (49–83)	0.109
Total bilirubine, mg/dl	0.88 (0.60–1.44)	0.76 (0.53–1.73)	1.05 (0.61–1.68)	0.265
tSOFA score, n	5.0 (2.5–6.0)	4.0 (2.0–5.0)	5.0 (3.5–6.0)	0.078
Neo/Cr, μmoL/mol	290 (183–563)	274 (171–436)	366 (231–632)	0.784
IL-8, pg/mL	6.3 (4.6–11.2)	6.4 (4.8–9.5)	6.3 (4.0–13.8)	0.362
IL-6, pg/mL	9.5 (3.5–25.2)	6.2 (2.7–15.5)	21.6 (9.6–28.0)	0.236
IL-6 ≥ 8.3, n (%)	21 (51)	8 (32)	13 (81)	0.004
Perioperative data				
Surgery time, min	325 (270–385)	310 (270–375)	333 (249–390)	0.961
CPB time, min	83 (74–102)	82 (74–107)	84 (71–99)	0.562
ACC time, min	46 (36–56)	49 (36–60)	46 (34–52)	0.436

Data are expressed as median and interquartile range (I-III) or number (percentage).

*ACC*, aortic cross-clamp; *ACEi*, angiotensin converting enzyme inhibitor*; ATII*, angiotensin II receptor antagonists; *CI*, cardiac index; *CPB*, cardiopulmonary by-pass; *IABP*, intraortic balloon pump; *IDC*, idiopathic dilated cardiomyopathy; *ICM*, ischemic cardiomyopathy; *INR*, International Normalized Ratio; *INTERMACS*, Interagency Registry for Mechanically Assisted Circulatory Support; *LVEDV*, left ventricular end-diastolic volume; *LVEDD*, left ventricular end-diastolic diameter; *LVEF*, left ventricular ejection fraction; *MAP*, mean arterial pressure; *NYHA*, New York Heart Association; *PCWP*, pulmonary capillary wedge pressure; *RAP*, right atrial pressure; *tSOFA*, total Sequential Organ Failure Assessment; WBC, white blood cells count.

Twenty-six (63%) patients were implanted with HeartMate II LVADs (Thoratec, Pleasanton, CA), 8 (20%) with De Bakey LVADs (MicroMed Technology, Houston, TX), 6 (15%) with Incor LVADs (Berlin Heart AG, Germany), and 1 (2%) with HeartWare LVAD (HeartWare, Framingham, MA).

**Figure 1:**
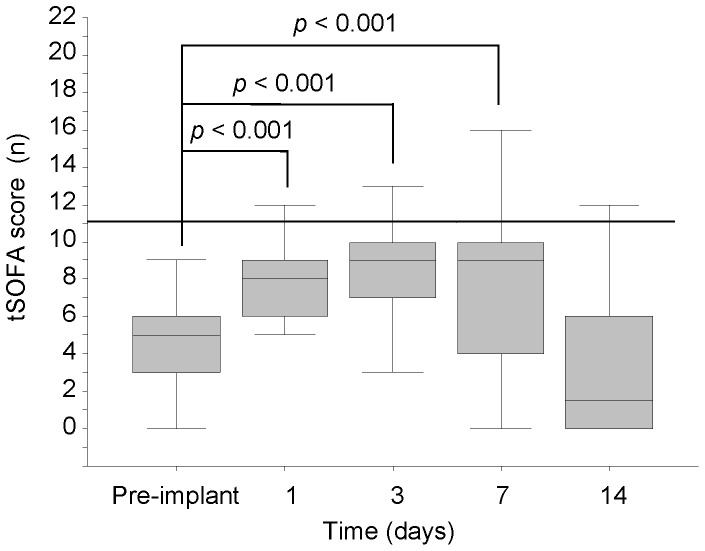
Postoperative tSOFA profile. Postoperative profile of tSOFA score in all LVAD-recipients. The tSOFA score ≥ 11 is pointed out by a dashed line.

After 1 week on MCS, hemodynamic improvement was observed in all patients with increase of cardiac index [1.7 (1.4–2.0) vs 3.0 (2.6–3.4) L/min/m^2^ at pre-implant and 1-week post-LVAD, respectively, *p*<0.001] and decrease of pulmonary capillary wedge pressure [26 (18–30) vs 9 (7–11) mmHg at pre-implant and 1-week post-LVAD, respectively, *p*<0.001]. Differently, tSOFA score was significantly increased 1 day after intervention with respect to pre-implant value (Figure 1), maintaining higher levels at 3 days and 1 postoperative week. At 2 postoperative weeks, tSOFA score was comparable to preoperative value (Figure 1).

During ICU stay 8 out of 41 (20%) LVAD-patients died of MOF, septic shock and esophageal haemorrhage [14 (11–23) days]. Detailed clinical in-hospital outcomes and primary causes leading to terminal MOF and death are summarised in Table 2. Among survivors, the length of ICU stay was of 13 (10–19) days, while hospitalisation was of 49 (42–77) days. Eight of the survivor patients experienced severe multi-organ failure evidenced by postoperative tSOFA score ≥11, mainly during the first postoperative week (Figure 1).

**Table 2:** Complications and causes of death “on-LVAD”

	All Cases	Group A	Group B	*P*
	(n = 41)	(n = 20)	(n = 21)	
**ICU Complications**				
Need of postoperative IABP	2 (5)	1 (5)	1 (5)	1.000
Bleeding				
Requiring surgery	4 (10)	2 (5)	2 (5)	1.000
Requiring > 2 PRBC units	34 (83)	15 (75)	19 (91)	0.238
Hemorrhagic	10 (24)	3 (15)	7 (33)	0.277
Embolism	1 (2)	-	1 (5)	1.000
Arrhytmias				
Atrial	12 (29)	6 (30)	6 (29)	1.000
Ventricular	4 (10)	1 (5)	3 (14)	0.606
Ventricular tachycardia	4 (10)	1 (5)	3 (14)	0.606
Infection				
Sepsis	3 (7)	1 (5)	2 (10)	1.000
Local non device-related infection	5 (12)	2 (10)	3 (14)	1.000
SIRS	3 (7)	-	3 (14)	0.232
Respiratory failure	13 (32)	4 (20)	9 (43)	0.181
Renal failure^a^	29 (71)	12 (60)	17 (81)	0.181
Hepatic dysfunction^b^	25 (61)	9 (45)	16 (76)	0.058
Right heart failure	23 (56)	7 (35)	16 (76)	0.012
Psychological	6 (15)	-	6 (29)	0.021
Other neurological	2 (5)	1 (5)	1 (5)	1.000
**ICU deaths**				
MOF	5 (12)	2 (10)	3 (14)	1.000
Esophageal haemorrhage	1 (2)	-	1 (5)	1.000
Septic shock	2 (5)	-	2 (10)	0.488

Values are presented as number (percentage).

*PRBC*, packed red blood cells; *SIRS*, systemic inflammatory response syndrome.

a Post eGFR < 60 ml/min/1.73 m^2^ or reduction of postoperative eGFR > 25% with respect to baseline.

b Post total bilirubine > 2 mg/dL and/or postoperative change of total bilirubine > 0.5 mg/dL with respect to baseline.

The pre-implant levels of IL-6, IL-8 and Neo/Cr of all LVAD-candidates were 9.5 (3.5–25.2) pg/mL, 6.3 (4.6–11.2) pg/mL and 290 (183–563) µmol/moL, respectively.

### Relationship between pre-implant cytokine levels and composite adverse outcome

Sixteen of 41 patients (39%) experienced postoperative tSOFA score ≥11 and/or ICU-death, together considered as composite critical outcome. Right heart failure, renal failure and hepatic dysfunction were the main complications contributing to the increased postoperative tSOFA score (Table 2).

**Figure 2:**
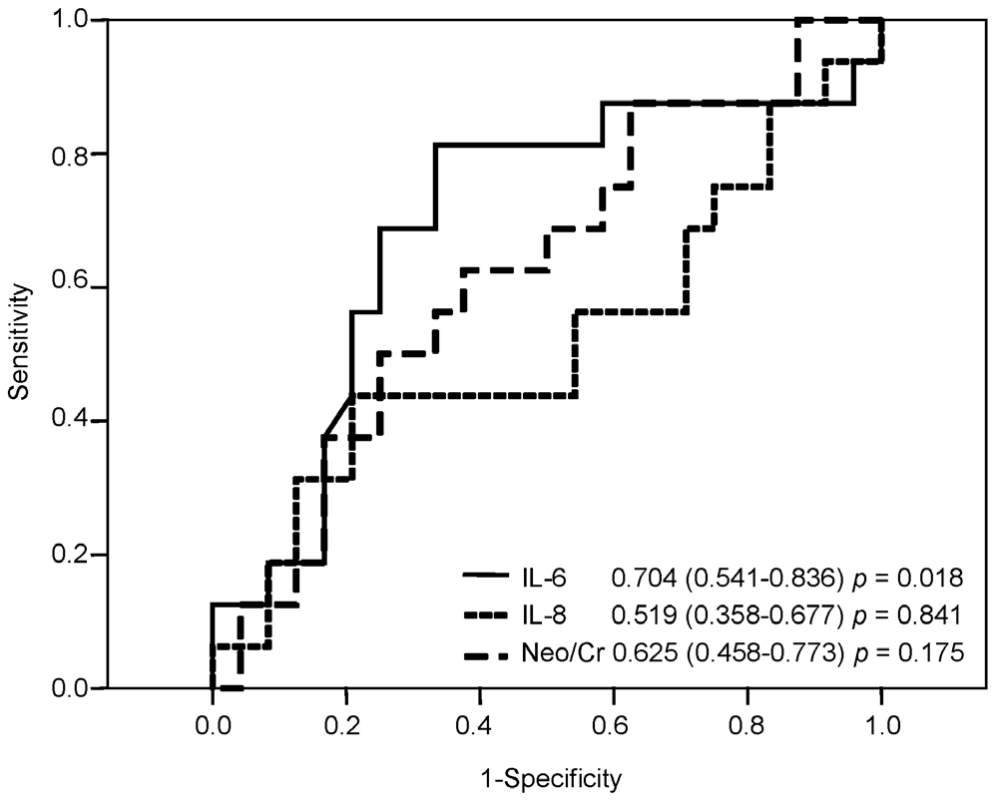
ROC curves of IL-6, IL-8 and neopterin. Receiver operating characteristic (ROC) curves of IL-6, IL-8 and Neo/Cr for the detection of patients with composite critical outcome in the early phase of LVAD support.

Among the ROC curve analysis for IL-6, IL-8 and Neo/Cr, pre-implant IL-6 levels were identified as the only significant marker for discrimination between patients with or without composite critical outcome (Figure 2); the ROC curve indicated an optimal cut-off-point for IL-6 at 8.3 pg/ml, with a sensitivity of 81% and a specificity of 68%.

By univariable analysis, pre-implant plasma IL-6 levels ≥ 8.3 pg/ml and right atrial pressure were significantly higher in LVAD-patients that experienced adverse composite outcome than in patients without composite outcome (Table 1). The tSOFA score was higher, but only as a trend, in LVAD-patients that experienced adverse composite outcome than in patients without composite outcome (Table 1). Surgery-related variables as well as type of used devices (not showed in the table) were comparable between groups.

The variables that reached the significance level of *p*<0.10 were entered into the final multivariable logistic regression analysis. The only parameter independently associated with composite outcome was pre-implant plasma IL-6 levels ≥ 8.3 pg/ml (OR 6.642, 95% CI 1.201–36.509, *p* = 0.030).

### Patient characteristics according to pre-implant IL-6 levels

Retrospectively, LVAD-candidates were divided in 2 groups according to pre-implant IL-6 cutoff of 8.3 pg/ml. Twenty patients with pre-implant IL-6 levels ≤ of 8.3 pg/ml were assigned to group A [3.5 (1.2–6.1) pg/mL, ranging from 0.4 to 8.3 pg/mL], while the other 21 patients with pre-implant IL-6 levels >8.3 pg/ml were assigned to group B [24.6 (16.4–38.0) pg/mL, ranging from 9.5 to 500.5 pg/mL]. Pre-implant IL-6 levels of all LVAD-candidates were higher than those observed in CHF patients [9.5 (3.5-25.2) and 2.3 (1.5–5.6) pg/mL, respectively, *p* = 0.002], but among LVAD candidates, only patients of group B showed IL-6 levels significantly higher than CHF patients (*p*<0.001). Detailed in-hospital complications and causes of death between A and B groups are described in Table 2.

The etiology was comparable between 2 groups of LVAD-candidates defined in according to pre-implant IL-6 cutoff of 8.3 pg/ml (Table 3). Echocardiographic parameters as well as medical therapies didn’t differ between the groups. Preoperative values of white blood cells (WBC) and tSOFA score were significantly higher in B- than in A-group. Among inflammatory variables, only Neo/Cr levels were higher, but only as a trend, in patients of B-group than in patients of A-group (Table 3).

**Table 3:** Univariable logistic regression analysis of variables associated to patient group with pre-implant IL-6 > 8.3 pg/mL.

	Group A	Group B	*P*
	(n = 20)	(n = 21)	
Age, yrs	55 (46–60)	55 (48–64)	0.446
Male gender, n (%)	18 (92)	19 (94)	0.959
Etiology, n (%)			0.164
IDC	10 (50)	15 (71)	
ICM	10 (50)	6 (29)	
NYHA class, n (%)			0.181
III	9 (43)	4 (20)	
IV	12 (57)	16 (80)	
INTERMACS, n (%)			
1	3 (15)	8 (38)	(Reference)
2	6 (30)	3 (14)	0.087
3+4	11 (55)	10 (48)	0.182
LVEF, %	22 (19–24)	20 (18–26)	0.925
LVEDV, ml	235 (185–315)	267 (188–317)	0.777
LVEDD, mm	70 (59–77)	69 (65–78)	0.852
CI, L/min/m^2^	1.70 (1.36–1.95)	1.67 (1.37–2.10)	0.736
RAP, mmHg	5 (3–9)	6 (5–10)	0.112
PCWP, mmHg	28 (20–33)	25 (16–29)	0.200
MAP, mmHg	75 (68–84)	78 (70–83)	0.789
Treatments, n (%)			
ACEi+ATII	15 (79)	14 (70)	0.524
Beta-Blocker	13 (77)	11 (55)	0.179
Statins	7 (41)	5 (25)	0.299
Diuretics	18 (95)	14 (70)	0.072
Inotropic	11 (55)	14 (67)	0.261
Inotropic equivalent, n	8 (2–10)	8 (4–11)	0.468
IABP, n (%)	5 (25)	8 (38)	0.370
INR	1.12 (1.03–1.29)	1.21 (1.14–1.47)	0.292
WBC, 10^9^/L	7.3 (6.1–8.7)	8.7 (7.9–12.0)	0.012
Lactate, nmol/l	1.00 (0.70–1.40)	1.10 (0.78–1.88)	0.192
eGFR, ml/min/1.73 m^2^	86 (57 –122)	79 (58–92)	0.238
Total bilirubine, mg/dl	0.69 (0.47–1.37)	1.31 (0.62–1.77)	0.115
tSOFA score, n	3.5 (3.0–4.8)	6.0 (4.0–7.0)	0.006
Neo/Cr, μmoL/mol	246 (136–295)	374 (252–693)	0.059
IL-8, pg/mL	6.2 (4.5–7.8)	10.9 (4.9–14.8)	0.088

Data are expressed as median and interquartile range (I-III) or number (percentage).

Group A: patients with pre-implant IL-6 levels ≤ of 8.3 pg/ml; Group B: patients with pre-implant IL-6 levels > 8.3 pg/ml. For abbreviations see Table 1.

The variables that reached the significance level of *p*<0.10 were entered into the final multivariable logistic regression analysis. The only parameter independently associated with pre-implant IL-6 levels higher than 8.3 pg/ml was WBC (OR 1.491, 95% CI 1.004–2.217, *p* = 0.048).

### Relationships with tSOFA score at 1 week, ICU stay, hospitalisation and 3-month survival according to pre-implant IL-6 levels

Pre-implant levels of cytokines were not significantly correlated to tSOFA score at 1 week (IL-6: R_s_ = 0.28, *p* = 0.077; IL-8: R_s_ = 0.15, *p* = 0.361; Neo/Cr: R_s_ = 0.05, *p* = 0.749). However, patients with pre-implant IL-6 levels >8.3 pg/ml showed higher tSOFA score at 1 week than patients with pre-implant IL-6 levels ≤ 8.3 [9 (8–10) and 5 (3–10), respectively, *p* = 0.030].

Among survivors, pre-implant IL-6 and IL-8 levels were significantly related to the length of ICU stay (IL-6: R_s_ = 0.52, *p* = 0.002; IL-8: R_s_ = 0.38, *p* = 0.028), and post LVAD hospitalisation (IL-6: R_s_ = 0.38, *p* = 0.028; IL-8: R_s_ = 0.42, *p* = 0.016).

**Figure 3:**
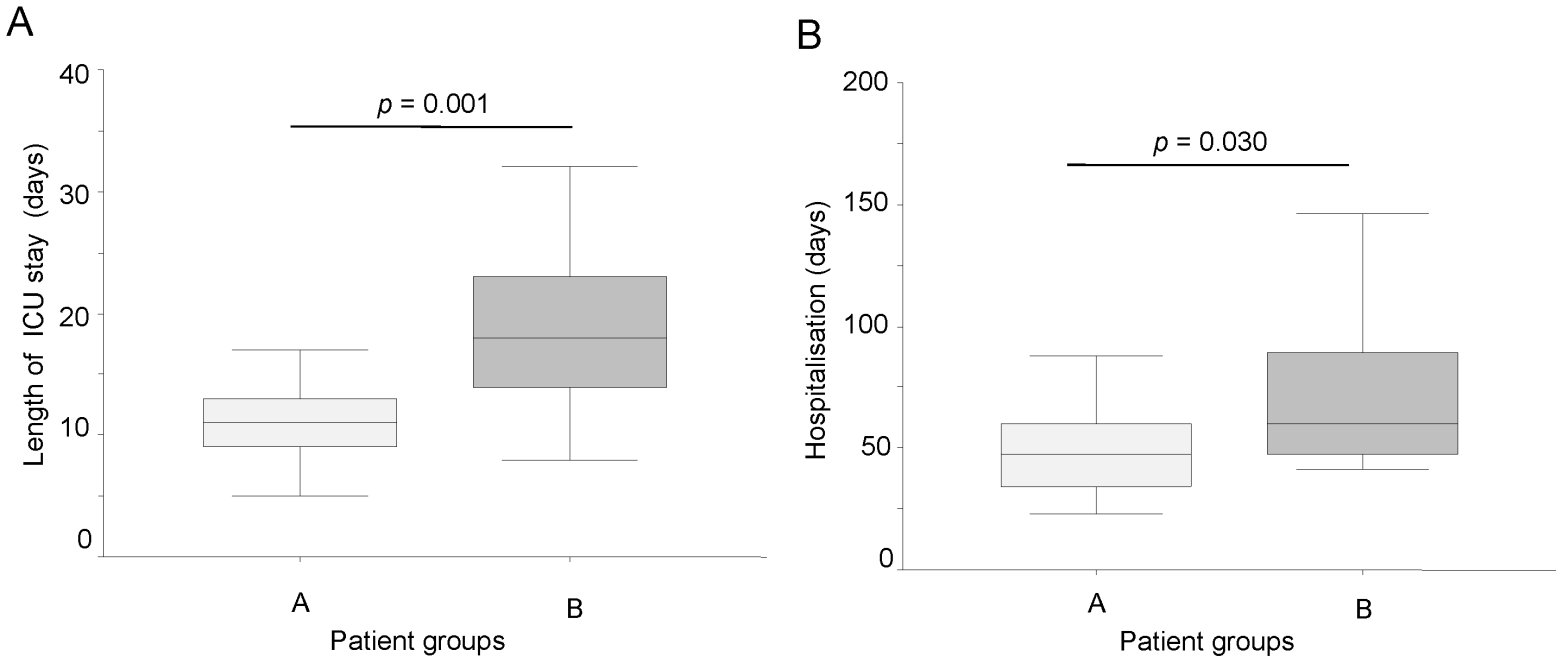
ICU stay and hospitalisation according to IL-6 levels. Length of ICU stay (A) and hospitalisation (B) in according to pre-implant IL-6 cutoff. A-group (empty box-plots): patients with plasma IL-6 levels ≤8.3 pg/ml; B-group (dark box-plots): patients with plasma IL-6 levels >8.3 pg/ml.

Patients with pre-implant IL-6 levels >8.3 pg/ml showed more prolonged ICU stay and hospitalisation than patients with pre-implant IL-6 levels ≤ 8.3 (Figure 3), with more frequent complications, in particular hepatic dysfunction and right heart failure (Table 2).

The 3-month survival rate was comparable with ICU survival rate (81%). The frequency of death was higher, although not significantly, in patients with pre-implant IL-6 levels > 8.3 pg/ml than patients with pre-implant IL-6 levels ≤ 8.3 [6 (30%) and 2 (10%) died patients, respectively, *p* = 0.238].

### Neopterin and cytokine profiles according to pre-implant IL-6 levels

**Figure 4:**
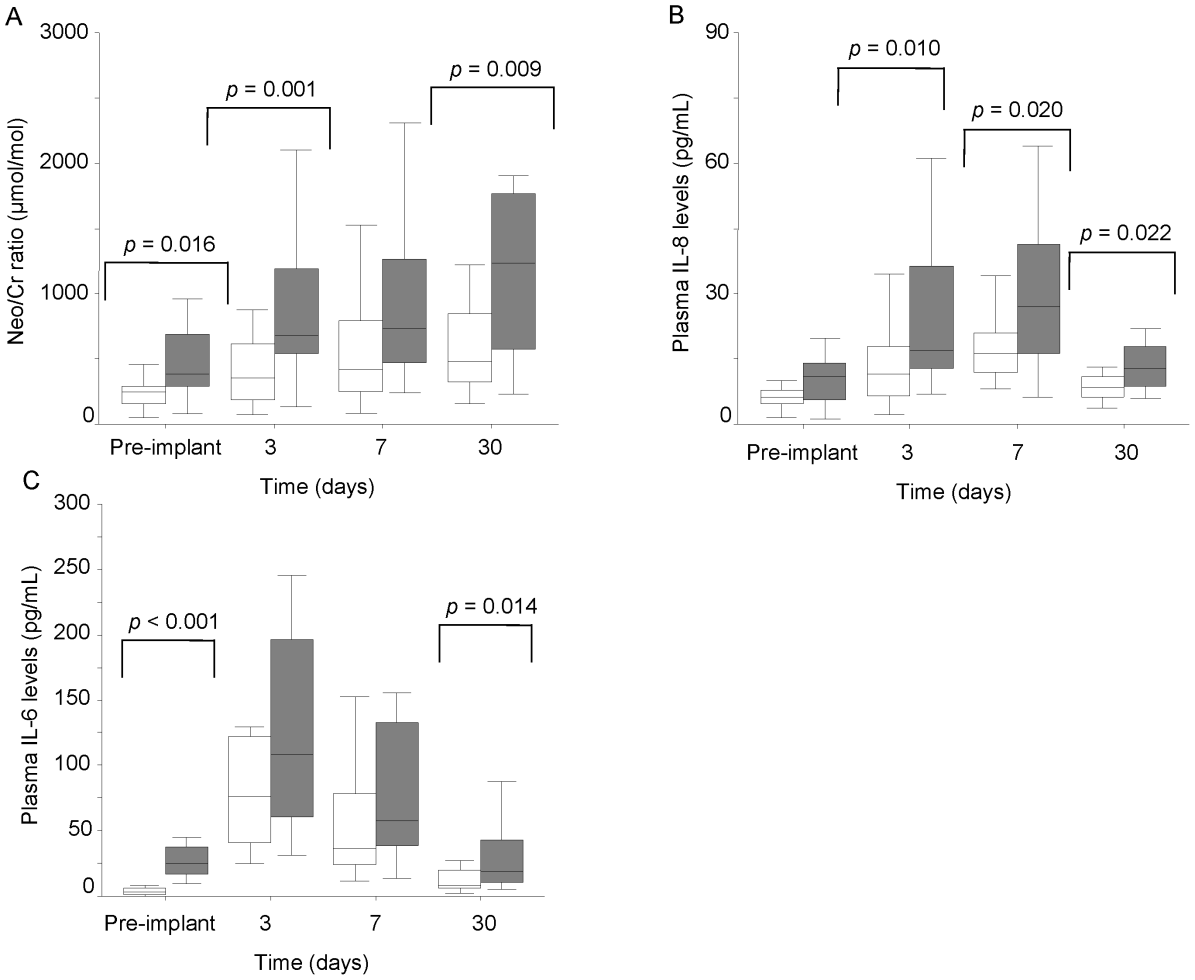
Postoperative inflammatory profiles. Postoperative profiles of neopterin (A), IL-8 (B) and IL-6 (C) according to pre-implant IL-6 cutoff (A-group, empty box-plots; B-group, dark box-plots).

The Neo/Cr levels progressively increased in both groups after LVAD implantation, but, at 3 days, Neo/Cr levels were significantly higher than baseline only in B-group (*p* = 0.002, Figure 4A). Moreover, postoperative levels of Neo/Cr were always higher in B- than in A-group (Figure 4A).

Likewise, also the IL-8 levels showed a progressive increment after device implantation in both groups compared to baseline values (Figure 4B); however, postoperative IL-8 levels were always higher in B- than in A-group (Figure 4B).

Differently, in both groups, the IL-6 profiles showed a peak at 3 days, higher than baseline (*p*<0.001 and *p* = 0.006 in A- and B-groups, respectively). In A-group, postoperative IL-6 levels maintained higher than baseline, also after 7 days and 1 month (*p*<0.001, both at 7 days and 1 month), while in B-group, the IL-6 levels at 7 days and 1 month were comparable to the baseline levels. However, at 1 month, the IL-6 levels were higher in B- than in A-group (Figure 4C).

## Discussion

The main findings of this study may be summarized as follows: 1) ESHF-patients supported by LVAD with preoperative IL-6 levels higher than 8.3 pg/mL are more susceptible of poor early outcome, longer ICU stay and hospitalisation, when compared to patients with lower IL-6 levels; 2) postoperatively, LVAD-patients with IL-6 levels higher than 8.3 pg/mL showed a more pronounced neopterin and IL-8 release, and MOF severity.

Recent advances in MCS, specifically implantable CF-LVAD therapy, are providing alternatives for patients waiting for heart transplantation (HT), for patients who are HT ineligible or anticipated to experience recovery after LV-unloading [1]-[4]. Every centre involved in advanced HF treatments has to evaluate patient specific risk profile according to one's own experience and to data reported by larger studies. With worsening of clinical status, the need for LVAD increases as well as the peri-operative risk, and optimal operative timing becomes difficult. In this setting, clinical indications, absolute or relative contraindications are not universally accepted because of contrasting published data.

With regard to risk stratification in ESHF-patients, little is known about baseline inflammatory profiles and their impact on clinical outcome and prognosis, and it's reasonable to speculate a role of inflammatory system on the outcome of these fragile patients. In the present study, pre-implant levels of IL-6, IL-8 and neopterin were investigated to evaluate the impact of these monocyte-related inflammatory mediators on the inflammatory response and outcome in LVAD patients. IL-8, a known chemokine attracting monocyte on endothelial cells [17], neopterin, a pteridine produced by activated macrophages [16], and IL-6-dependent signals, mainly associated to progression of HF, are proposed as crucial triggers in controlling monocyte activation and recruitment in vascular inflammation and endothelial dysfunction, important factors for development of MOF [10], [18]. Moreover, neopterin is a key pteridine that links inflammation and redox state in heart failure. Indeed macrophages, stimulated by interferon-gamma, generate neopterin that interferes with reactive species, such as peroxynitrite, inducing myocardial contractile failure [19]. However, in our cohort of LVAD-candidates, only patients with preoperatively elevated IL-6 levels, particularly higher than 8.3 pg/ml, were more susceptible to experience serious complications, as severe MOF, with postoperative tSOFA score ≥11, and/or death in ICU, independently from IL-8 and neopterin levels, as well as from the amount of the pre-implant multi-organ dysfunction. Indeed, in critically ill patients, differences in mortality have been previously reported to be better predicted by the maximal t-SOFA score in the first days of ICU stay; tSOFA score higher than 10 has been associated with elevated mortality rates [15]. Moreover, in our series, patients with elevated IL-6 levels were also characterised by a longer ICU stay, hospitalisation and higher tSOFA score after 1 week, reflecting a greater disarrangement of multi-organ function than in those with lower IL-6 levels. Altogether, these data suggest a more critical clinical course in patients with preoperative elevated IL-6 levels than in patients with lower IL-6 levels.

The concentration range of IL-6 levels has been found extremely broad in our LVAD-candidates, ranging from negligible to extremely pathological values, greater than the highest value found in CHF patients. These data suggest that in a few ESHF patients, the hemodynamic collapse requiring LVAD implantation is associated with increased activation of systemic inflammation, linked to the IL-6 signals; among preoperative variables, IL-6 levels are associated only with the total leukocyte count, regardless of the hemodynamic status, as defined by INTERMACS profiles. Therefore, the evaluation of IL-6 levels in LVAD-candidates may provide additional information on patient's risk profile, in addition to the prognostic information provided by the INTERMACS profiles, and could allow to highlight patients more susceptible of poorer outcome in the early phase of LVAD support, although not strictly associated to the risk of death. Indeed, in our series of patients, the pre-implant cut-off-point for IL-6 at 8.3 pg/ml did not allow to predict survival in the short-time (3 months) of LVAD support.

Postoperatively, elevated IL-6 levels were reported in patients who died because of MOF in the early phase of LVAD support, and the activation of monocytes was proposed as a crucial mechanism involved in the development of MOF [7]. In a previous study we reported that, after LVAD implantation, neopterin levels progressively increased mainly in non-survivors [6]. In the present cohort, postoperative Neo/Cr and IL-8 levels increased mainly in patients who showed preoperative IL-6 levels higher than 8.3 pg/ml, reflecting, postoperatively, a more marked monocyte activation and adverse inflammatory milieu. Moreover, postoperative IL-6 levels showed similar profiles in both groups, with a peak level in the first postoperative days. This finding supports the hypothesis that only IL-6-dependent inflammatory signals, present at pre-implant, may be responsible for triggering stimuli that favor a more marked monocyte activation and adverse inflammatory milieu after LVAD implantation, as evidenced by the greater release of IL-8 and neopterin. In addition, the greater neopterin release in patients with preoperative elevated IL-6 levels might reflect a more marked pro-oxidant behavior, since neopterin is also capable of enhancing peroxynitrite production, favoring LDL oxidation, that exerts chemotactic properties on macrophages [20]. Therefore, different ranges of IL-6 levels in ESHF-patients needing a LVAD support, might differently affect the redox processes and immune response to stress stimuli succeeding LVAD implantation, thus influencing the clinical course and early outcome.

Kirsh et al. [21] reported that a low percentage of monocytes expressing HLA-DR molecules, during the immediate phase of device support, was predictive of ICU-death, suggesting that a low percentage of HLA-DR positive monocytes reflects a postoperative immunoparalysis that hampers tissue repair processes necessary for end-organ recovery. HLA-DR expression is reported as a phenotypic marker of functional monocyte deactivation, making controversial clinical interpretation of the monitoring of neopterin in LVAD-patients. However, the concomitant presence of reduced proportions of CD14^+^ HLA-DR cells with elevated levels of neopterin was reported in trauma patients and sepsis, together proposed as biomarkers reflecting an immune response, not balanced, susceptible to favors sepsis and adverse MOF [22]–[24]. Therefore, the elevated levels of neopterin and IL-8 found in our LVAD-patients with a poorer outcome might reflect an altered monocyte-mediated immune response, influenced by pre-implant IL-6 levels.

Our single centre study was limited by its relatively small number of patients; the results are not related to a single device but to different CF-LVADs. However, the findings of this study underscore the importance to consider the inflammatory parameters related with monocyte activation during the decision making process of ESHF-patients, to deepen the knowledge of clinical features of patients and better stratify the operative risk, and the risk of MOF or death after LVAD implantation.

Finally, preoperative elevated IL-6 levels, higher than 8.3 pg/mL, are associated, after intervention, to higher release of markers related with the monocyte activation, prolonged course and poorer outcome. Further studies in larger population are needed to validate the cut-off value of IL-6 and of other potential biomarkers which could be helpful in targeting the most appropriate treatment.

## References

[1] Lund LH, Matthews J, Aaronson K (2010) Patient selection for left ventricular assist devices. Eur J Heart Fail 12: 434–443. 20172939 10.1093/eurjhf/hfq006

[2] Dickstein K, Cohen-Solal A, Filippatos G, McMurray JJ, Ponikowski P, et al. ESC Committee for Practice Guidelines (CPG) (2008) ESC Guidelines for the diagnosis and treatment of acute and chronic heart failure 2008. The task force for the diagnosis and treatment of acute and chronic heart failure 2008 of the European Society of Cardiology. Developed in collaboration with the Heart Failure Association of the ESC (HFA) and endorsed by the European Society of Intensive Care Medicine (ESICM). Eur J Heart Fail 10: 933–989. 18826876 10.1016/j.ejheart.2008.08.005

[3] Hunt SA, Abraham WT, Chin MH, Feldman AM, Francis GS, et al (2009) American College of Cardiology Foundation; American Heart Association (2009) 2009 focused update incorporated into the ACC/AHA 2005 Guidelines for the Diagnosis and Management of Heart Failure in Adults: a report of the American College of Cardiology Foundation/American Heart Association Task Force on Practice Guidelines: developed in collaboration with the International Society for Heart and Lung Transplantation. Circulation 119: e391–e479. 19324966 10.1161/CIRCULATIONAHA.109.192065

[4] Wilson SR, Mudge GH, Stewart Jr GC, Givertz MM (2009) Evaluation for a ventricular assist device: selecting the appropriate candidate. Circulation 119: 2225–2232. 19398678 10.1161/CIRCULATIONAHA.109.850610

[5] Shiga T, Kinugawa K, Hatano M, Yao A, Nishimura T, et al (2011) Age and preoperative total bilirubin level can stratify prognosis after extracorporeal pulsatile left ventricular assist device implantation. Circ J 75: 121–128. 21116070 10.1253/circj.cj-10-0770

[6] Caruso R, Trunfio S, Milazzo F, Campolo J, De Maria R, et al (2010) Early expression of pro- and anti-Inflammatory cytokines in left ventricular assist device recipients with multiple organ failure syndrome. ASAIO J 56: 313–318. 20445439 10.1097/MAT.0b013e3181de3049

[7] Masai T, Sawa Y, Ohtake S, Nishida T, Nishimura M, et al (2002) Hepatic dysfunction after left ventricular mechanical assist in patients with end-stage heart failure: role of inflammatory response and hepatic microcirculation. Ann Thorac Surg 73: 549–555. 11845873 10.1016/s0003-4975(01)03510-x

[8] Deng MC, Edwards LB, Hertz MI, Rowe AW, Keck BM, et al (2005) International Society for Heart and Lung Transplantation (2005) Mechanical circulatory support device database of the International Society for Heart and Lung Transplantation: Third Annual Report-2005. J Heart Lung Transplant 24: 1182–1187. 16143231 10.1016/j.healun.2005.07.002

[9] Caruso R, Verde A, Cabiati M, Milazzo F, Boroni C, et al (2012) Association of pre-operative interleukin-6 levels with Interagency Registry for Mechanically Assisted Circulatory Support profiles and intensive care unit stay in left ventricular assist device patients. J Heart Lung Transplant 31: 625–633. 22386451 10.1016/j.healun.2012.02.006

[10] Wiel E, Vallet B, ten Cate H (2005) The endothelium in intensive care. Crit Care Clin 21: 403–416. 15992664 10.1016/j.ccc.2005.03.001

[11] Nieminen MS, Bohm M, Cowie MR, Drexler H, Filippatos GS, et al (2005) ESC Committee for Practice Guideline (CPG). Executive summary of the guidelines on the diagnosis and treatment of acute heart failure: The Task Force on Acute Heart Failure of the European Society of Cardiology. Eur Heart J 26: 384–416. 15681577 10.1093/eurheartj/ehi044

[12] Minne L, Abu-Hanna A, de Jonge E (2008) Evaluation of SOFA-based models for predicting mortality in the ICU: a systematic review. Crit Care 12: R161. 19091120 10.1186/cc7160 PMC2646326

[13] Palardy M, Nohria A, Rivero J, Lakdawala N, Campbell P, et al (2010) Right ventricular dysfunction during intensive pharmacologic unloading persists after mechanical unloading. J Card Fail 16: 218–224. 20206896 10.1016/j.cardfail.2009.11.002 PMC3913073

[14] Levey AS, Greene T, Kusek JW, Beck GJ (2000) Simplified equation to predict glomerular filtration rate from serum creatinine. J Am Soc Nephrol 11: 828A.10770960

[15] Ferreira FL, Bota DP, Bross A, Mélot C, Vincent JL (2001) Serial evaluation of the t-SOFA score to predict outcome in critically ill patients. JAMA 286: 1754–1758. 11594901 10.1001/jama.286.14.1754

[16] De Rosa S, Cirillo P, Pacileo M, Petrillo G, D’Ascoli GL, et al (2011) Neopterin: from forgotten biomarker to leading actor in cardiovascular pathophysiology. Curr Vasc Pharm 9: 188–199. 10.2174/157016111794519372 21143176

[17] Castellani ML, De Lutiis MA, Toniato E, Conti F, Felaco P, et al (2012) Impact of RANTES, MCP-1 and IL-8 in mast cells. J Biol Regul Homeost Agents 24: 1–6. 20385066

[18] Brasier AR (2010) The nuclear factor-kB–interleukin-6 signalling pathway mediating vascular inflammation. Cardiovasc Res 86: 211–218. 20202975 10.1093/cvr/cvq076 PMC2912657

[19] Ferdinandy P, Danial H, Ambrus I, Rothery RA, Schulz R (2000) Peroxynitrite is a major contributor to cytokine-induced myocardial contractile failure. Circ Res 87: 241–247. 10926876 10.1161/01.res.87.3.241

[20] Herpfer I, Greilberger J, Ledinski G, Widner B, Fuchs D, et al (2002) Neopterin and 7,8-dihydroneopterin interfere with low density lipoprotein oxidation mediated by peroxynitrite and/or copper. Free Radic Res 36: 509–520. 12150539 10.1080/10715760290025898

[21] Kirsch M, Boval B, Damy T, Ghendouz S, Vermes E, et al (2012) Importance of monocyte deactivation in determining early outcome after ventricular assist device implantation. Int J Artif Organs 35: 169–176. 22461111 10.5301/ijao.5000053

[22] Walsh DS, Thavichaigarn P, Pattanapanyasat K, Siritongtaworn P, Kongcharoen P, et al (2005) Characterization of circulating monocytes expressing HLA-DR or CD71 and related soluble factors for 2 weeks after severe, non-thermal injury. J Surg Res 29: 221–230. 10.1016/j.jss.2005.05.003 16045935

[23] Ploder M, Pelinka L, Schmuckenschlager C, Wessner B, Ankersmit HJ, et al (2006) Lipopolysaccharide-induced tumor necrosis factor alpha production and not monocyte human leukocyte antigen-DR expression is correlated with survival in septic trauma patients. Shock 25: 129–134. 16525350 10.1097/01.shk.0000191379.62897.1d

[24] Muller Kobold AC, Tulleken JE, Zijlstra JG, Sluiter W, Hermans J, et al (2000) Leukocyte activation in sepsis; correlations with disease state and mortality. Intensive Care Med 26: 883–892. 10990102 10.1007/s001340051277

